# Visual impairment, age-related eye disease, and sleep dysfunction in older adults

**DOI:** 10.1038/s41433-025-03777-3

**Published:** 2025-04-12

**Authors:** Alan Y. Huang, Joshua R. Ehrlich, Ali G. Hamedani

**Affiliations:** 1 Departments of Neurology, Ophthalmology, and Epidemiology, Perelman School of Medicine, University of Pennsylvania, Philadelphia, PA USA; 2 Department of Ophthalmology and Visual Sciences, University of Michigan, Ann Arbor, MI USA; 3 Survey Research Center, Institute for Social Research, University of Michigan, Ann Arbor, MI USA

**Keywords:** Epidemiology, Visual system, Eye diseases, Risk factors, Neurological disorders

## Abstract

**Background/Objectives:**

The visual system affects circadian rhythms, and both visual and sleep difficulties are common in older adults. This study examines the association between visual impairment, age-related eye disease, and sleep disturbances among older adults in the United States.

**Subjects/Methods:**

This cross-sectional study used Round 11 of the National Health and Aging Trends Study (NHATS). Vision was assessed using self-report and objective assessments (distance and near acuity, contrast sensitivity). Medicare claims data were used to identify diagnoses of age-related macular degeneration, glaucoma, diabetic retinopathy, and cataract. Primary outcomes included self-reported sleep disturbances, defined by difficulties in sleep initiation, maintenance, and medication use. Logistic regression models were adjusted for demographic and clinical variables.

**Results:**

Among 3817 participants (56% female), difficulty with sleep initiation, maintenance, and medication use were reported by 41.7%, 44.2%, and 26.5% of the cohort, respectively. In unadjusted models, self-reported visual difficulty was associated with sleep initiation (OR 1.80, 95% CI: 1.43–2.29) and maintenance difficulties (OR 1.53, 95% CI: 1.16–2.02) and sleep medication use (OR 1.68, 95% CI: 1.27–2.24). After adjusting for covariates, self-reported visual difficulty remained significantly associated with sleep medication use (OR 1.40, 95% CI: 1.00–1.95). Near acuity and contrast sensitivity were associated with sleep initiation difficulties but did not remain significant after adjustment. No associations were found between ophthalmic diagnoses and outcomes.

**Conclusion:**

Self-reported visual difficulty is associated with increased sleep medication use in older adults. Because visual impairment and sleep medications are associated with falls and cognitive decline, future studies should consider these comorbidity patterns.

## Introduction

Vision is a key regulator of sleep and circadian rhythms. The capture and transmission of light to the hypothalamus through the retinohypothalamic tract stimulates the release of hormones such as cortisol and melatonin, which control sleep-wake cycles to generate the endogenous 24-h circadian rhythm [1, 2]. This connection between the visual and circadian systems is exemplified by the prevalence of sleep-wake rhythm disorders among people who are totally blind [3]. Light is also used as a therapy for certain sleep disorders, serving as a natural experiment for the role of visual input in sleep [2]. However, the mechanisms and directionality of the relationship between vision and sleep as a whole remain incompletely understood [4], particularly in older adults, a population in which both visual impairment and sleep dysfunction are common and contribute to impaired quality of life [5, 6]. Previous studies have examined the association between self-reported visual difficulty and sleep duration cross-sectionally [7–10], but studies that consider the effects of different age-related eye diseases on other aspects of sleep are lacking [11–13]. In this study, we examined the relationship between self-reported and performance-based measures of visual function, diagnosed age-related eye disease, and self-reported sleep dysfunction using data from the National Health and Aging Trends Study (NHATS), a longitudinal, nationally representative sample of Medicare beneficiaries in the U.S [14, 15].

## Methods

This study was approved by the University of Pennsylvania Institutional Review Board (ID #852882). Informed consent was previously obtained at the time of study enrollment and was not separately required for this secondary analysis. The study was adherent to the Declarations of Helsinki.

### Data

We used data from NHATS, a nationally representative sample of Medicare beneficiaries ages 65 and older who have been surveyed annually since 2011, with replenishment of the sample in 2015 [14, 15]. NHATS collects information on physical and cognitive function as well as a variety of survey measures and social determinants of health through a combination of telephone interviews and in-home visits [15]. In 2021 (round 11), this included measurements of near and distance visual acuity and contrast sensitivity as described below [16]. The Medicare program is the primary insurer for 97% of the U.S. population ages 65 and above, and Medicare linkage is available for all NHATS participants during each year of survey participation and up to five years prior to survey entry. We used the Master Beneficiary Summary File (MBSF) to obtain Medicare enrollment and eligibility information and the carrier file to identify ophthalmic diagnoses and procedures.

### Primary exposures: visual impairment and age-related eye disease

Visual impairment was defined using both self-reported and performance-based measures. Self-reported visual difficulty was defined if the participant reported being blind or unable to perform any of the following activities: read newspaper print, recognize someone across the street, or watch TV from across a room. Vision was also measured using distance and near visual acuity and contrast sensitivity using tablet-based tests with habitual correction (i.e. glasses or contact lenses). We defined impairment in distance or near visual acuity as a logarithm of the minimum angle of resolution (logMAR) of greater than 0.30 (equivalent to worse than 20/40 Snellen acuity), which is in accordance to the World Health Organization’s thresholds for vision impairment as defined in the ICD-11 [17]. Impaired contrast sensitivity was defined as log contrast sensitivity (logCS) less than 1.55, which represents two standard deviations below the mean of a normative sample [18].

In addition to self-reported and performance-based vision, we considered the following age-related eye diseases: primary open-angle glaucoma (POAG, which included normal/low-tension glaucoma), age-related macular degeneration (AMD), diabetic retinopathy (DR), and cataract. We selected these diseases because they are relatively common among older adults, thereby providing maximal statistical power [19]. Using ICD-10-CM codes in the 2019-2021 Medicare carrier file (Supplementary Table 1 in the Appendix), we considered an individual to be diagnosed with a given eye disease if they received two or more diagnoses by an ophthalmologist (provider code 18) or optometrist (provider code 41) on different dates. If someone with a previous cataract diagnosis had a claim for cataract extraction or pseudophakia prior to the NHATS round 11 survey visit, they were recorded as not having cataract at the time of analysis. For each eye disease, we identified a corresponding control group. Controls consisted of all subjects who had no recorded diagnosis and had complete Part A and B eligibility (including at least one claim in the carrier file) in 2019, 2020, and 2021.

### Primary outcomes: sleep disturbances

The NHATS questionnaire included three questions to assess sleep initiation, maintenance, and medication use, respectively: how often it takes more than 30 min to fall asleep at night; how often a person had trouble falling back asleep on nights they woke up; and how often they took medication to sleep [14]. Sleep initiation, maintenance, and medication use were analyzed separately, and we compared subjects who reported experiencing each outcome two or more times a week to those who experienced them once a week or less frequently, consistent with previous studies [20, 21].

### Covariates

The following variables were included as covariates in our analysis: age, gender, race/ethnicity, education level, body mass index (BMI), hypertension, diabetes, heart attack or heart disease, stroke, lung disease, cancer, depression, anxiety, self-reported pain, and dementia. BMI was calculated from self-reported height and weight. Depression and anxiety were measured in NHATS using the Patient Health Questionnaire for Depression and Anxiety (PHQ-4), a validated screening tool with detailed methods described previously [22]. We dichotomized clinically significant depressive and anxiety symptoms using a cutoff score of 3 for both depression and anxiety subscales of the PHQ-4, which yields the highest sensitivity and specificity for both major depressive disorder and generalized anxiety disorder [23, 24]. Self-reported pain was coded as no pain, non-limiting pain, and limiting pain based on participant responses to questions asking if they have been bothered or limited by pain in the past month. To ascertain dementia, NHATS includes questions about self-reported cognitive difficulties (e.g., trouble remembering appointments or handling finances), physician diagnoses of dementia, and in-person cognitive testing (e.g., orientation, delayed recall, clock drawing). These items are combined into a validated algorithm that classifies dementia status as “probable dementia”, “possible dementia”, or “no dementia”. For this study, we defined dementia as a rating of “possible dementia” or “probable dementia” [25].

### Statistical analysis

We used descriptive statistics to summarize the baseline characteristics of NHATS respondents with and without sleep disturbances and logistic regression models to evaluate the association between visual impairment, age-related eye disease, and sleep difficulties while adjusting for covariates. All models accounted for the complex survey design of NHATS, and variance estimation was performed using modified balanced repeated replication (BRR) as recommended by NHATS [26]. Statistical analyses were performed using Stata/SE 18 (College Station, TX), and statistical significance was defined as two-sided *p* < 0.05. Depression and anxiety are known risk factors for sleep difficulties, but there is also evidence that visual impairment is associated with depression and anxiety, so these may lie on a causal pathway between vision and sleep. We therefore performed sensitivity analyses in which we adjusted for all of our prespecified covariates except for depression and anxiety.

## Results

We analyzed data from 3817 participants in NHATS round 11. The most common age group was 75–79 (32.2%), and 56% were female. Difficulty with sleep initiation, maintenance, and medication use were reported by 41.7%, 44.2%, and 26.5% of the cohort, respectively. Baseline characteristics for each outcome are summarized in Table 1.

**Table 1:** Baseline characteristics and prevalence of sleep disturbances in older adults.

	Total population (*n* = 3817)	Sleep initiation difficulty		Sleep maintenance difficulty		Sleep medication use	
		Yes (*n* = 1439, 41.7%)	No (*n* = 1921, 58.3%)	Yes (*n* = 1488, 44.2%)	No (*n* = 1866, 55.8%)	Yes (*n* = 843, 26.5%)	No (*n* = 2529, 73.5%)
**Age**							
70–74	445 (28.1%)	182 (26.7%)	259 (29.4%)	173 (26.3%)	267 (29.9%)	116 (28.5%)	325 (28.1%)
75–79	987 (32.2%)	410 (32.5%)	569 (32.4%)	440 (32.8%)	538 (32.2%)	244 (31.8%)	735 (32.4%)
80–84	847 (20.0%)	375 (20.9%)	453 (19.3%)	368 (20.2%)	460 (19.8%)	216 (21.2%)	617 (19.6%)
85–89	651 (11.9%)	268 (12.4%)	356 (11.5%)	281 (12.6%)	342 (11.3%)	148 (11.0%)	478 (12.3%)
90+	536 (7.7%)	204 (7.6%)	284 (7.3%)	226 (8.1%)	259 (6.8%)	119 (7.6%)	374 (7.5%)
**Female**	2250 (56.0%)	948 (63.5%)	1016 (50.4%)	938 (59.5%)	1019 (52.8%)	547 (61.4%)	1424 (53.9%)
**Race/ethnicity**							
White	2717 (80.9%)	935 (76.3%)	1462 (84.1%)	1023 (78.0%)	1368 (83.1%)	631 (82.6%)	1773 (80.2%)
Black	770 (8.2%)	360 (10.3%)	317 (6.6%)	321 (8.8%)	355 (7.6%)	134 (6.4%)	546 (8.8%)
Other	92 (3.7%)	40 (4.5%)	38 (2.9%)	40 (4.7%)	39 (2.7%)	23 (3.8%)	57 (3.6%)
Hispanic	184 (7.2%)	83 (8.9%)	81 (6.4%)	79 (8.4%)	85 (6.6%)	44 (7.2%)	120 (7.4%)
**Education**							
Less than high school	664 (14.2%)	299 (17.5%)	234 (9.9%)	289 (16.6%)	245 (10.3%)	144 (14.8%)	394 (12.5%)
High school/some college	1777 (47.5%)	730 (52.3%)	859 (44.5%)	721 (48.8%)	866 (47.0%)	414 (49.3%)	1178 (47.1%)
Bachelor/ associate	728 (22.0%)	242 (19.2%)	434 (24.7%)	270 (21.0%)	401 (23.4%)	167 (22.7%)	510 (22.2%)
Master/ professional	556 (16.3%)	149 (11.0%)	371 (20.9%)	186 (13.6%)	334 (19.3%)	110 (13.2%)	413 (18.2%)
**BMI**							
<18.5	79 (2.0%)	33 (2.0%)	44 (2.0%)	35 (1.9%)	39 (1.9%)	19 (2.2%)	59 (1.9%)
18.5–24.9	1147 (32.2%)	459 (30.6%)	671 (32.9%)	480 (31.7%)	650 (32.2%)	298 (34.1%)	842 (31.5%)
25–29.9	1190 (36.8%)	492 (35.0%)	695 (38.5%)	520 (35.6%)	666 (38.1%)	282 (36.4%)	904 (37.0%)
≥30.0	902 (29.0%)	420 (32.4%)	479 (26.7%)	421 (30.8%)	478 (27.7%)	224 (27.3%)	676 (29.6%)
**Depression**	889 (23.8%)	536 (35.1%)	337 (15.2%)	507 (31.6%)	364 (17.0%)	306 (32.9%)	575 (20.3%)
**Anxiety**	706 (20.2%)	439 (29.6%)	256 (13.2%)	461 (30.9%)	236 (11.5%)	269 (29.7%)	433 (16.7%)
**Hypertension**	2612 (73.4%)	1158 (76.1%)	1433 (71.5%)	1189 (74.8%)	1396 (72.2%)	664 (74.4%)	1935 (73.0%)
**Diabetes**	1038 (29.6%)	501 (33.1%)	524 (27.0%)	492 (32.5%)	536 (27.3%)	266 (30.0%)	763 (29.3%)
**Heart attack/disease**	975 (25.4%)	466 (29.0%)	498 (22.6%)	489 (28.8%)	478 (22.6%)	294 (30.9%)	675 (23.4%)
**Stroke**	80 (2.3%)	36 (2.2%)	42 (2.2%)	42 (2.8%)	35 (1.7%)	23 (2.5%)	57 (2.2%)
**Lung disease**	822 (23.4%)	407 (28.3%)	407 (19.9%)	404 (26.7%)	407 (20.7%)	251 (27.3%)	568 (22.0%)
**Cancer**	199 (6.0%)	86 (6.1%)	113 (6.0%)	94 (6.2%)	105 (6.0%)	56 (6.7%)	142 (5.8%)
**Pain**							
No pain	1477 (44.3%)	512 (35.0%)	958 (51.0%)	533 (35.9%)	932 (50.9%)	280 (33.6%)	1192 (48.2%)
Non-limiting pain	858 (25.2%)	390 (27.0%)	464 (24.0%)	381 (24.8%)	471 (25.6%)	210 (26.2%)	646 (24.9%)
Limiting pain	1041 (30.5%)	535 (38.0%)	495 (25.0%)	573 (39.2%)	458 (23.5%)	351 (40.2%)	684 (26.9%)
**Dementia**							
No dementia	2666 (84.1%)	1100 (81.2%)	1565 (87.1%)	1173 (83.4%)	1490 (85.7%)	657 (83.2%)	2008 (84.7%)
Probable or possible	722 (15.9%)	339 (18.8%)	356 (12.9%)	315 (16.6%)	376 (14.3%)	186 (16.8%)	521 (15.3%)

Counts are unweighted, but column frequencies incorporate NHATS sample weights.

**Fig. 1:**
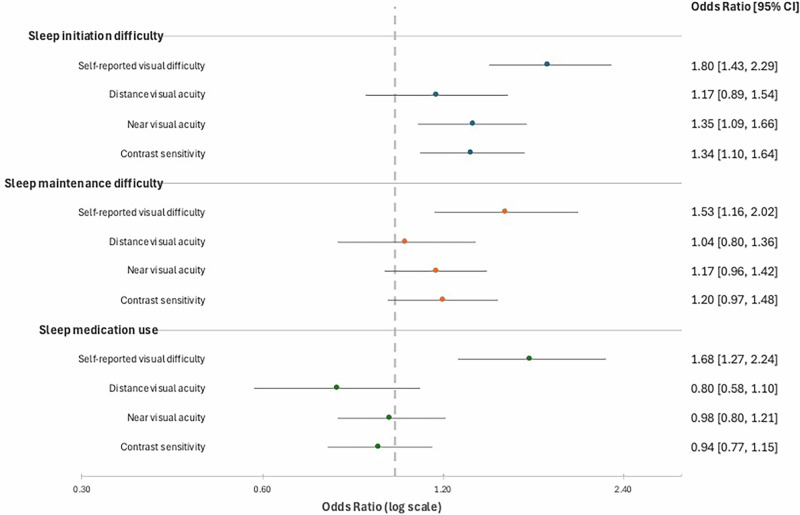
Crude association between visual difficulty and sleep disturbances in older adults.

**Fig. 2:**
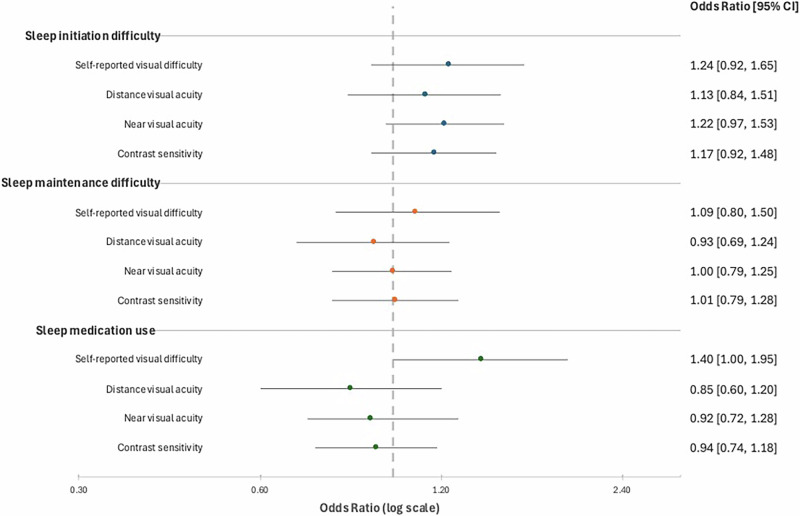
Multivariable adjusted association between visual difficulty and sleep disturbances in older adults.

In unadjusted logistic regression models, self-reported visual difficulty was associated with increased odds of having sleep initiation difficulties (OR 1.80, 95% CI: 1.43–2.29), sleep maintenance difficulties (OR 1.53, 95% CI: 1.16–2.02), and sleep medication use (OR 1.68, 95% CI: 1.27–2.24) (Fig. 1). After adjusting for covariates, self-reported visual difficulty remained significantly associated with increased odds of sleep medication use (OR 1.40, 95% CI: 1.00–1.95) but not sleep initiation or maintenance measures (Fig. 2). In unadjusted logistic regression models, near visual acuity impairment (OR 1.35, 95% CI 1.09–1.66) and reduced contrast sensitivity (OR 1.34, 95% CI 1.10–1.64) were associated with increased odds of having sleep initiation difficulties (Fig. 1). However, these attenuated after covariate adjustment, and performance-based measures of visual function were not associated with sleep maintenance or medication use (Fig. 2). There was no association between POAG, AMD, DR, or cataract and sleep initiation, maintenance, or medication use (Table 2).

**Table 2:** Association between age-related eye disease and sleep disturbances in older adults.

	Sleep initiation difficulty			Sleep maintenance difficulty			Sleep medication use		
	Prevalence (*n*, %)	Unadjusted OR (95% CI)	Adjusted OR (95% CI)	Prevalence (n, %)	Unadjusted OR (95% CI)	Adjusted OR (95% CI)	Prevalence (*n*, %)	Unadjusted OR (95% CI)	Adjusted OR (95% CI)
**AMD** (*n* = 727)	286 (40.07%)	1.02 (0.81–1.29)	0.85 (0.65–1.10)	328 (45.70%)	1.20 (0.94–1.52)	1.06 (0.80–1.42)	195 (28.22%)	1.10 (0.82–1.50)	0.99 (0.68-1.45)
**POAG** (*n* = 764)	332 (42.41%)	1.13 (0.86–1.47)	1.03 (0.76–1.41)	349 (46.39%)	1.19 (0.92–1.54)	1.08 (0.80–1.46)	202 (27.93%)	1.06 (0.83–1.35)	1.09 (0.81-1.46)
**DR** (*n* = 196)	94 (46.05%)	1.37 (0.91–2.07)	0.86 (0.52–1.43)	95 (49.40%)	1.35 (0.88–2.05)	1.04 (0.58–1.88)	51 (25.15%)	0.91 (0.64–1.27)	0.74 (0.45-1.20)
**Cataract** (*n* = 690)	280 (38.24%)	0.88 (0.69–1.12)	0.92 (0.70–1.23)	292 (43.55%)	1.02 (0.80–1.31)	1.07 (0.83–1.37)	169 (26.11%)	0.97 (0.73–1.28)	0.96 (0.70-1.32)

Counts are unweighted, percentages and regression coefficients incorporate NHATS sample weights.

*OR* odds ratio, *AMD* age-related macular degeneration, *POAG* primary open-angle glaucoma, *DR* diabetic retinopathy.

In sensitivity analyses that did not adjust for depression and anxiety due to their potential existence on a causal pathway between vision and sleep, self-reported visual difficulties were associated with sleep initiation (OR 1.44, 95% CI: 1.09–1.92) and medication use (OR 1.54, 95% CI: 1.10–2.15), and near acuity was associated with sleep initiation (OR 1.26, 95% CI: 1.01–1.56). The rest of our findings were unchanged (Supplementary Table 3).

## Discussion

In this study, we examined the association between self-reported and performance-based vision, age-related eye disease, and sleep disturbances in a nationally representative sample of older U.S. adults. We found that individuals with self-reported visual difficulty were significantly more likely to take medication for sleep. Other measures of vision and sleep were not associated with one another after covariate adjustment in our primary analyses, but in sensitivity analyses, self-reported and performance-based measures of vision were associated with sleep initiation. Because sleep medication use carries a significant risk of falls and other adverse outcomes in older adults, these findings highlight the vulnerabilities and comorbidities associated with visual impairment.

We found that self-reported visual impairment was associated with 40% greater odds of sleep medication use after adjusting for confounders. This is consistent with previous findings from a large health survey in Sweden, which also found that self-reported visual impairment was associated with sleep medication use [27]. Sedative and hypnotic medications which are used to promote sleep are associated with an increased risk of falls and cognitive decline, and many are included in the Beers Criteria of potentially inappropriate medications for adults over 65 years of age [28, 29]. There is growing evidence that self-reported visual impairment may also be a risk factor for both falls and cognitive decline in older adults [5, 30, 31]. It is therefore possible that sleep medication use may interact with or mediate some of the relationship between visual impairment and these outcomes. Because medication use and prescription patterns are modifiable through public service announcements, deprescribing initiatives, and other public health interventions, this should be explored in future studies. The fact that self-reported visual difficulty was associated with sleep medication use but not with sleep initiation or maintenance difficulties may indicate that medication use is a proxy for more severe difficulty with sleep. Alternatively, individuals with visual impairment may experience sleep difficulties with a similar frequency but are more likely to receive medication for them because of differences in healthcare access and delivery.

In addition to self-reported vision, NHATS began collecting performance-based measures of vision in round 11, providing a more clinically oriented perspective on vision in aging [16]. Using these data, we found that near visual acuity impairment and reduced contrast sensitivity were associated with sleep initiation difficulties in unadjusted analyses, and in our sensitivity analyses, near acuity remained significantly associated with sleep initiation. These clinical findings are important because poor sleep itself may exacerbate cognitive decline or reduce quality of life in older adults—factors that are particularly detrimental for individuals already managing visual difficulties [32]. The lack of association between other performance-based measures of vision and sleep maintenance or medication use highlights the discordance between self-reported and performance-based measures of vision in both research and clinical practice, as well as potential biases introduced by self-reported metrics such as overreporting of impairments or differences in recall of visual difficulties [33].

Using linked Medicare claims data, we found that diagnoses of AMD, POAG, DR, and cataract were not significantly associated with sleep outcomes. Some previous studies have identified associations between these eye diseases and sleep disturbances, but results have been mixed [1, 11, 34, 35], and in case-control studies where patients with a specific eye disease (e.g., AMD) are compared to healthy controls [11], the identification of controls from a clinic-based convenience sample rather than the general population may predispose to selection bias. Our use of a nationally representative, population-based sample mitigates this issue and may explain some of the discrepancies between our findings and prior results. Moreover, studies that rely solely on administrative claims data (e.g. Medicare) are limited to capturing only those sleep disturbances that result in healthcare encounters [36]. Many individuals with sleep difficulties may not seek medical care, which could lead to underreporting of sleep problems and bias the associations with visual impairment [37]. In contrast, the NHATS survey asks all participants about their sleep habits, thus reducing this potential bias. Lastly, much of the previous literature has focused on obstructive sleep apnea, a condition we did not specifically examine [13, 38].

Our study took a multifaceted approach to visual impairment that incorporated self-reported and performance-based measures as well as diagnosed eye disease. However, we were limited to three self-reported questions about sleep, and while self-reported sleep is a major determinant of quality of life, it may not correlate with performance-based measures of sleep latency, maintenance, and duration that are obtained from polysomnography or wearable sensors. NHATS also did not use a validated sleep survey, which could have included data on other aspects of sleep such as quality, fragmentation, and duration. Other limitations include the cross-sectional nature of our analysis, the lack of adequate indicators of ocular disease severity using ICD-10 codes, and the potential for residual confounding or type I error in our analyses of self-reported vision and sleep medication use. Our sensitivity analyses also highlight the importance of distinguishing between confounders and mediators, and the fact that depression and anxiety may act as both complicates studies of vision and health outcomes such as cognitive impairment and sleep. Despite these limitations, our study is unique in its statistical power and use of performance-based visual measures to ascertain associations between vision and sleep. Future studies could leverage wearable technology as an accessible research tool to collect objective measures of sleep disturbances in a longitudinal manner. Additionally, further research should be conducted to assess the relationship of comorbidities such as falls and cognitive decline.

## Supplementary information

Supplemental Material

## Data Availability

NHATS public use files are available for download through the NHATS website. Access to the restricted CMS data used in this research is available by obtaining appropriate data use agreements from NHATS and the NIA Data Linkage Program.
