# Supplementary material for: Visual impairment, age-related eye disease, and sleep dysfunction in older adults
Source: Eye (Lond). 2025 Apr 12;39(10):2008–14. doi: 10.1038/s41433-025-03777-3 (PMC12209426; doi:10.1038/s41433-025-03777-3)
Supplement: Supplementary file 1 — Supplemental Material [file 41433_2025_3777_MOESM1_ESM.docx]

**Supplemental Table 1: Ophthalmic diagnosis and procedure codes**

| Diagnosis | ICD-10-CM codes | HCPCS (CPT) codes |
| --- | --- | --- |
| Primary open-angle glaucoma | H4000* H4001* H4002* H4005*  H4010* H4011*  H4012* H4015* | N/A |
| Age-related macular degeneration | H3530 H3531* H3536* H3532* | N/A |
| Diabetic retinopathy | E083* E093* E103* E113* E123* E133* | N/A |
| Cataract | H25* H26* H28* | N/A |
| Pseudophakia/aphakia | Z961 Z984* H270* |  |
| Cataract extraction | N/A | 66830 66840 66850  66852 66920 66930  66940 66982 66983  66984 |

**Supplemental Table 2: Procedure codes and algorithms to identify eye care utilization in Medicare claims data**

| At least one of the following CPT codes: | OR | At least one of the following CPT codes plus provider code 14 or 41 |
| --- | --- | --- |
| 65850 65855 65865 65870 65880 65900 65930 66150 66155 66160 66165 66170 66172 66180 66184 66185 66500 66505 66600 66625 66630 66700 66710 66720 66740 66761 66762 67036 67038 67039 67040 67101 67105 67107 67108 67109 67110 67112 67208 67210 67218 67227 67228 67220 67221 76511 76512 76513 76516 92002 92004 92012 92014 92018 92019 92020 92081 92082 92083 92100 92120 92130 92140 92225 92226 92230 92235 92240 92250 92260 92275 92283 92284 92287 |  | 99024 99025 99201 99202 99203 99204 99205 99211 99212 99213 99214 99215 99241 99242 99243 99244 99245 99251 99252 99253 99254 99255 99261 99262 99263 99271 99272 99273 99274 99275 99281 99282 99283 99284 99285 |

**Supplemental Table 3:** **Sensitivity analysis of association between visual difficulty and sleep disturbances in older adults without adjusting for depression and anxiety**

| Outcome | Visual difficulty category | Odds ratio (95% CI) |
| --- | --- | --- |
| Sleep initiation difficulty | Self-reported visual difficulty | **1.44 (1.09-1.92)** |
|  | Distance visual acuity | 1.16 (0.85-1.59) |
|  | Near visual acuity | **1.26 (1.01-1.56)** |
|  | Contrast sensitivity | 1.23 (0.98-1.56) |
| Sleep maintenance difficulty | Self-reported visual difficulty | 1.28 (0.96-1.70) |
|  | Distance visual acuity | 0.98 (0.74-1.30) |
|  | Near visual acuity | 1.05 (0.85-1.31) |
|  | Contrast sensitivity | 1.09 (0.85-1.38) |
| Sleep medication use | Self-reported visual difficulty | **1.54 (1.10-2.15)** |
|  | Distance visual acuity | 0.85 (0.61-1.19) |
|  | Near visual acuity | 0.95 (0.74-1.22) |
|  | Contrast sensitivity | 0.98 (0.78-1.24) |

Odds ratios are adjusted for age, gender, race/ethnicity, education level, body mass index (BMI), hypertension, diabetes, heart attack or heart disease, stroke, lung disease, cancer, self-reported pain, and dementia.
